# Calcium arrhythmogenicity of Purkinje fibers: importance of the animal model

**DOI:** 10.3389/fphys.2025.1676701

**Published:** 2025-10-30

**Authors:** Bruno D. Stuyvers

**Affiliations:** Faculty of Medicine–Division of Biomedical Sciences–Memorial University of Newfoundland, St. John’s, NL, Canada

**Keywords:** calcium, arrhythmia, vt, VF, Purkinje fiber, Purkinje cell, myocardial ischemia, myocardial infarction

## Abstract

Ventricular tachycardias (VTs) and fibrillations (VFs) are frequent complications of ischemic myocardial infarction (MI). Because their initiation mechanism remains unknown, these arrhythmias are virtually unpredictable and often degenerate into cardiac arrest and syncope without immediate medical assistance. Electrical mapping and ablation techniques have located the origin of ischemic arrhythmias in the terminal arborizations of the cardiac conduction system, the Purkinje fibers. A classical model of MI in the dog has demonstrated that abnormal calcium (Ca^2+^) cycling in the Purkinje cells (Pcells) is the source of non-driven depolarizations (DADs) in the conduction tissue and is likely to create the pro-arrhythmic conditions of human ischemic heart. A better understanding of Ca^2+^ abnormalities in Pcells post infarction is an evident prerequisite for elucidating the mechanism of ischemic arrhythmias. Nevertheless, a unique Ca^2+^ handling system was discovered in Pcells, exhibiting fundamental differences compared with the well-known model of Excitation-Contraction coupling of ventricular cardiomyocytes. This cellular specificity of Purkinje fibers was observed in large mammalian species but not in murine hearts, where Purkinje cells are comparable to ventricular myocytes and designed to respond to 400–600 stimulations/min. The present report reviews the mechanism of Ca^2+^ arrhythmogenicity in Pcells of large mammalian hearts and documents the need for animal models that simulate the size and function of human hearts to study ischemic arrhythmias.

## Introduction

Sudden cardiac death (SCD) accounts for 15%–20% of the mortality in adults worldwide. This dramatic issue is most frequently associated with ischemic heart disease (or coronary artery disease) and is caused by life-threatening ventricular tachycardia (VTs) and fibrillation (VFs). These tachyarrhythmias commonly arise in the setting of myocardial ischemia secondary to a coronary occlusion, particularly in the early stage of the myocardial infarction (MI) (Haissaguerre et al., 2016). The pathophysiology of those arrhythmias involves a complex interplay between ischemia-induced changes in ion channel function, cellular metabolism, and structural remodelling in the ventricular myocardium (Ter Keurs and Boyden, 2007). However, the Purkinje fibers have been increasingly recognized as key contributors to the specific arrhythmogenesis post-MI (Haissaguerre et al., 2016; Nogami, 2011; Benali et al., 2024). There is a consensual agreement that this arrhythmic risk originates from intracellular calcium dysregulation in Purkinje cells (Ter Keurs and Boyden, 2007). Intracellular Ca^2+^ concentration oscillations in these cells generate non-driven (non-sinusal) electric impulses in the fibers, which trigger focal ectopic activity and reentry mechanisms in the myocardium. Understanding these Ca^2+^ abnormalities in Purkinje cells is the key to identifying and treating a fundamental cause of SCD post myocardial infarction. As with most human diseases, animal models are indispensable for investigating the underlying mechanisms of arrhythmia. Nevertheless, many questions regarding the physiology of Purkinje fibers still make it difficult to choose a suitable model. Even though our understanding of Purkinje cells is incomplete, it already indicates that the classical model of cardiac “excitation-contraction” coupling (Bers, 2002) does not apply to those cells. In addition, numerous observations have demonstrated that Purkinje cells exhibit structural differences that result in their Ca^2+^ handling varying across species. Considering the consensual implication of Ca^2+^ in the triggered arrhythmias of ischemic heart (see (Ter Keurs and Boyden, 2007) for review), the choice of animal models is crucial for investigating the origins of ischemic arrhythmias post infarction in human patients. This brief review summarizes our current knowledge of Purkinje cells and highlights some key elements in selecting the most suitable animal model for the pathophysiology of human Purkinje fibers.

## Structural and electrophysiological characteristics of Purkinje cells

### The Purkinje fibers

**FIGURE 1:**
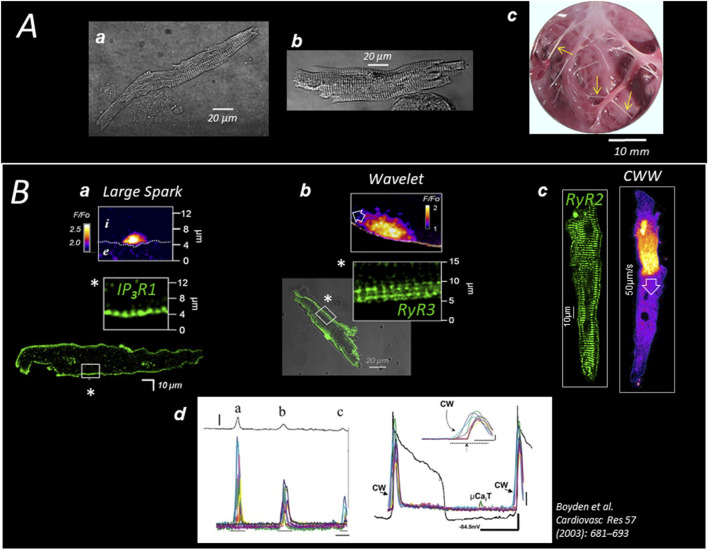
Specific Ca^2+^ handling of cardiac Purkinje cells in large mammalian species **(A)** Pig (a) and human (b) Purkinje cells are enzymatically dispersed from sub-endocardial (free-running) Purkinje strands (c; see arrows in pig heart). **(B)** The expression of 3 different channels (here in dog heart) delimits three concentric regions of SR-Ca^2+^ release; the first region (a) extends 2–3 µm under the sarcolemma (SL) and expresses the Inositol Phosphate Receptor *IP3R1* which generates large Ca^2+^ sparks; the second layer (b) extends 5–10 µm under the SL and expresses the (non-cardiac) ryanodine receptor *RyR3*; the RyR3 region produces small waves (Wavelets) which propagate on short distances exclusively at the cell periphery and are of the same amplitude than sparks; the third layer (c) fills most of the cell core, expressing the typical cardiac ryanodine receptor *RyR2* which produces large cell-wide waves (CWWs); CWWs (d) induce membrane depolarizations (DADs) proportional to the density and amplitude of the waves present in the cell or in the cell aggregate (left panel) and, occasionally, can trigger a full action potential (right panel).

As initially described by Sunao Tawara (Akiyama, 2010), the Purkinje fibers (Figure 1A) form the terminal arborizations of the conduction system, branching in every region of the ventricles as prolongations of the His-Purkinje bundles. They are responsible for transmitting the nodal impulses to the working cardiomyocytes and coordinating the contraction of the ventricular chambers during the heartbeat. The Purkinje tissue is present in the ventricles as free-running strands in the subendocardial region and transmural fibers in the endocardium (Pauziene et al., 2017; Boyden et al., 1989). To date, no evidence has been found to support fundamental differences between the two types of fibers. However, the ratio of free strands to transmural varies among species. This ratio determines the accessibility of these fibers in animal models, such as the rat, which has a high density of intramural fibers, or the dog, which has a high density of free strands in the subendocardial region. The fibers are connected to the myocardium by transitional cells (Martinez-Palomo et al., 1970). Although very little is known about transitional cells, a possible role in the regulation of the conduction at the Purkinje-myocardium interface has been proposed, and some studies have implicated these cells as potential sources of arrhythmogenicity (Blackwell et al., 2022; Behradfar et al., 2014).

### The cardiac Purkinje cells: structure and morphology

Purkinje cells and ventricular cardiomyocytes share the same rod shape and sarcomeric striation due to an organized myofibrillar system (Figure 1A). Although their density and arrangement in Purkinje cells vary among species (see Table 1), the myofibrils give both cell types a similar macroscopic appearance. This explains why the Purkinje cells are still referred to as “Purkinje myocytes” and have been considered a model for cardiac cell physiology in many past studies. In both cell types, the sarcomeres shorten when the surrounding Ca^2+^ concentration rises, but with apparently slower kinetics in Purkinje cells, suggesting differences in the protein composition of the sarcomeres. The reasons Purkinje cells encompass a functional contractile machinery remains unclear. It could be to coordinate the position of the Purkinje and myocardial fibers during the contraction.

**TABLE 1:** This table summarizes the comparative morphology and ultrastructure of cardiac Purkinje cells across several mammalian species.

Species	Cell morphology and size (Comparison vs. human)	Intercalated disks (IDs) and connexins (Cx)	Ultrastructure	References
Mouse	Small, cylindrical; length <80 µm; diameter 10 μm; (smaller than human)	Simple (staircase) IDs with few gap junctions; mainly Cx40, minimal Cx43, rare Cx45	Few myofibrils, Abundant SR (junctional); Normal T-tubules (vs. ventricular myocytes)	Ono et al. (2009), Miquerol et al. (2010)
Rat	Cylindrical, short bundles; length <120 μm, diameter <10 μm; (smaller than human)	Short (staircase) IDs with moderate gap junctions; mainly Cx40, low Cx43, rare Cx45	Few myofibrils; Abundant SR (junctional) Normal T-tubules (vs. ventricular myocytes)	Ono et al. (2009), Matsushita et al. (1999), Di Maio et al. (2007)
Rabbit	Cylindrical, elongated; Length 120 Diameter ∼20 µm (smaller than human)	disks distinct (limited data) Cx40 present, Cx43 minimal	Moderate, Peripheral myofibrils Well-developed SR (junctional); Limited to abundant T-tubules	Sommer and Johnson (1968), Cordeiro et al. (2001)
Cat	Cylindrical, branched, elongated; Length ∼120 µm Diameter ∼15 µm (slightly shorter and thinner than human)	disks distinct (limited data) Cx40 high (likely), Cx43 moderate	Moderate, peripheral myofibrils Well-developed SR (junctional and corbular); Limited to abundant T-tubules	Sommer and Johnson (1968)
Dog	Cylindrical or fusiform; length 120–180 μm, diameter 20–40 μm; (slightly shorter and thinner than human)	Large, complex (finger-like) IDs; abundant Cx40, moderate Cx43, minor Cx45	Abundant, peripheral myofibrils; Developed SR (junctional and corbular) T-tubules parse/discontinuous	Boyden et al. (1989), Sommer and Johnson (1968), Kanter et al. (1993)
Sheep	Cylindrical or Oval; length 150–220 μm, diameter 20–30 μm; (similar diameter but shorter than human)	Well-developed (finger-like) IDs; abundant Cx40, moderate Cx43, minor Cx45	Peripheral, abundant myofibrils Developed SR (junctional and corbular) T-tubules sparse	Mobley and Page (1972), Oliphant and Loewen (1976)
Pig	Cylindrical or Oval; length 150–200 μm, diameter 25–35 μm; (slightly shorter and thinner than human)	Complex (finger-like) IDs; abundant Cx40, moderate Cx43, minor Cx45	Peripheral, abundant myofibrils; Developed SR (junctional and corbular) T-tubules sparse	Garcia-Bustos et al. (2017), Daniels et al. (2017), Stankovičová et al. (2003)
Cow	Polygonal; length 180–250 μm, diameter 25–40 μm; (similar diameter but longer than human)	Numerous IDs; abundant Cx40, moderate Cx43, minor Cx45	Sparse myofibrils; Developed SR (corbular) T-tubules sparse	Ono et al. (2009), Forsgren and Thornell (1981)
Human	Cylindrical/fusiform; length 120–200 μm, diameter 30–50 µm	Well-developed (finger-like) IDs; abundant Cx40, moderate Cx43, minor Cx45	Peripheral abundant myofibrils; Developed SR (junctional and corbular) T-tubules sparse/discontinuous	Sommer and Johnson (1968), Anderson et al. (2013)

As shown in Table 1, the morphology of Purkinje cells varies among mammalian species, but the major differences are observed between small and large hearts, likely due to distinct electrophysiological constraints (see Table 2).

**TABLE 2:** Comparative Electrophysiology of Cardiac Purkinje Cells Across Mammals.

Species	RMP (mV)	AP amp (mV)	dV/dt_max (V/s)	APD50/APD90 (ms)	CV (m/s)	Automaticity	Major ionic currents	Refs
Mouse	≈ −70 to −80	Large AP amplitude (∼80–100)	Higher than ventricular myocytes	APD longer than ventricular myocytes	0.5–2	Spontaneous pacemaker activity observed in some isolated PCells	INa density larger than VMs; ICa,L and ICa,T present; Ito reduced; IK1 similar to VMs	Vaidyanathan et al. (2013)
Rabbit	≈ −75 to −85 (Purkinje strands)	∼80–110	∼150–300 higher than ventricular myocytes	APD90 often ∼200–400 ms at 1 Hz in tissue studies	∼1–2.5 multi- cellular prep	Uncommon *in situ*; more frequent in isolated cells	INa robust; ICaL present; Ito and Ikr present; contributions from ICaT variable	Cordeiro et al. (1998)
Dog	≈ −80 to −90 isolated canine Purkinje fibers	∼90–110	∼150–300 High upstroke velocities	APD90 200–400 ms at 1 Hz (long plateau)	0.8–2.5	Can show automaticity in isolated preparations; implication in arrhythmias	Prominent INa; ICaL present; lower Ito	Dun and Boyden (2008), Saitoh et al. (1989), Reder et al. (1981)
Sheep	∼ −80 to −90	AP amplitude large; exact values vary	high dV/dt and long APDs; no precise numeric values	APD90 often long (hundreds of ms)	1.5–3	Spontaneous activity occasionally observed	Major currents: INa, ICaL prominent; lower Ito vs. myocardium	Verkerk et al. (1999)
Pig	≈ −80 to −90	AP amplitude large (∼90–110 mV)	dV/dt_max high; numerical values vary with prep	APD50/APD90 long (hundreds of ms) in pig PF preparations at 1 Hz	∼1.5–3	Automaticity less common *in situ*; isolated cells may show pacemaker activity	Prominent INa, ICaL; Ito lower; connexin Cx40 expression strong	Garcia-Bustos et al. (2017), Gettes and Surawicz (1968), Dosdall et al. (2007)
Human	≈ −80 to −90 (varies with disease and sample)	∼80–110	>100	long APDs; APD90 ∼100	∼1–2.5	can show automaticity in isolated prep and are implicated in clinical arrhythmias	INa prominent; ICaL present; human PF vs. VM large differences	Dangman et al. (1982), Ideker et al. (2009)

Parameters: RMP, resting membrane potential; AP amp, action potential amplitude; dV/dt_max, maximum upstroke velocity; APD50/APD90, action potential duration at 50%/90% repolarization; CV, conduction velocity; Refs, References.

### The cardiac Purkinje cells: electrophysiology

The impulse velocity in the fibers varies with the heart size, likely related to the propagation distance, ranging from 1–2 m/s in small rodents to more than 4 m/s in larger animals (see Table 2), i. e., approximately tenfold larger compared to myocardium (0.3–0.4 m/s) (Durrer et al., 1970). The low resistance of Purkinje fibers (compared to myocardium) facilitates the rapid conduction of nodal impulses across the ventricle. Gap junctions in the intercalated disks participate in this low resistance. Electric and Ca^2+^ signals propagate cell-to-cell in the Purkinje fiber through the gap junctions, which, like those in ventricular myocytes, contain channels composed of connexins 40 (Cx40) and 43 (Cx43) (Table 1). The conductance of the Cx40 channel is twice as high as that of the Cx43 channel, and Cx40 is three times more concentrated in Purkinje cells compared to ventricular cells (Sivagangabalan et al., 2014; Kanter et al., 1993). This predominance of C x 40 is likely to facilitate the rapid intercellular current flow and contribute to the high conduction velocity in the Purkinje fibers. Connexin 45 forms low-conductance gap junction channels and is also expressed in the Purkinje cells, possibly modulating the conduction in the fibers (Dun and Boyden, 2008).

At the cellular level, presumably still related to the conduction function of Purkinje tissue, there are significant differences in the electrophysiology of Purkinje cells compared to ventricular myocytes, as thoroughly reviewed in (Dun and Boyden, 2008; Boyden et al., 2010). In brief, the Purkinje cells have a longer action potential with a prominent phase 1 of repolarization, longer APD50 and APD90, a more negative plateau, and larger AP amplitude than ventricular cells (Dun and Boyden, 2008). Although two levels of resting membrane potential (RMP) have been reported in Purkinje fibers (Gadsby and Cranefield, 1977), it is widely recognized that, under normal physiological conditions, the RP (∼-80 mV) is comparable to that of ventricular myocytes (Boyden et al., 2010). However, a slow diastolic depolarization due to the presence of I_F_ current can arise in Purkinje fibers in the absence of overdrive suppression by the sinus rhythm (Dun and Boyden, 2008). Although the regular activity of Purkinje fibers in the heart is triggered, the presence of I_F_ current attributes the Purkinje tissue with a natural tendency towards automaticity, ranging from 20 to 40 beats per minute in large mammalian species, including humans (Vassalle, 1977).

### Ca^2+^ handling and Ca^2+^ mobilization of Purkinje cells

The primary function of Purkinje cells is to generate an AP in response to evoked depolarization from adjacent cells in the fiber. The membrane depolarization and formation of action potential in Purkinje cells involve voltage-gated channels, with specific isoform profiles explicitly expressed in those cells as listed in (Dun and Boyden, 2008). Nearly as a side effect of the electrical transmission function of Purkinje cells, the depolarization is accompanied by an influx of Ca^2+^ in the cytosol due to the activation of two voltage gated Ca^2+^ channels: the L-type Ca^2+^ channel (LTCC) with the two isoforms Cav1.2 and Cav1.3, and the T-type Ca^2+^ channel (TTCC) with the three isoforms Cav3.1, Cav3.2, and Cav3.3 (Dun and Boyden, 2008; Rosati et al., 2007). Unlike ventricular myocytes, a large representation of T-type Ca^2+^ current (ICaT) compared to L-type Ca^2+^ current (ICaL) has been reported in Purkinje fibers (Tseng and Boyden, 1989). ICa,T activates at more hyperpolarizing potentials than the high-voltage ICa,L, which is predominant in cardiomyocytes. Strongly expressed in nodal cells and Purkinje cells, a role in pacemaker activity has been attributed to ICa,T (Mangoni and Nargeot, 2008). These Ca^2+^ currents trigger further Ca^2+^ release from the intracellular Ca^2+^ compartment, the sarcoplasmic reticulum (SR), in a process called “Ca^2+^ induced Ca^2+^ Release” (CICR) (Fabiato, 1983). In ventricular cardiomyocytes, the SR is arranged around the myofibrils. Tubular invaginations of the sarcolemma, called transverse tubules (T tubules), extend deep in the cell at the level of every Z-disc in the myofibrils. In this region, referred to as the “dyadic cleft”, the T-tubules are close to the terminal cisternae of the SR (junctional SR) so that L-type Ca^2+^ channels in the membrane of the tubules face clusters of Ca^2+^ channels, RyR2, in the SR membrane. This arrangement is ubiquitously distributed in cardiomyocytes and is crucial for the uniform CICR and synchronous contraction of those cells. Ca^2+^ is released from the junctional SR in the dyadic cleft, but it can also occur outside, from isolated extremities of the SR called corbular SR (Franzini-Armstrong et al., 1999).

In large mammalian species, such as dogs, sheep, pigs, and humans (see Table 1), Purkinje cells are devoid of organized transverse tubular system (Sommer and Johnson, 1968; Cordeiro et al., 2001; Daniels et al., 2017; Sommer and Johnson, 1970) and exhibit an internal structure comparable to that of atrial myocytes (Bootman et al., 2011; Mackenzie et al., 2004). In this condition, the intracellular Ca^2+^ mobilization in Purkinje cells primarily relies on the release of Ca^2+^ by the corbular SR in the core and, to a lesser extent, by the junctional SR under the membrane (Ter Keurs and Boyden, 2007). Upon electric stimulation, Ca^2+^ enters the cell mostly across the peripheral membrane through the voltage-gated inward Ca^2+^ currents. The absence of T tubules in the core predicts that, in Purkinje cells, the influence of Na-Ca exchange (NCX) is limited to a restricted space under the sarcolemma, known as the “SubSL” compartment (Stuyvers et al., 2005), and Ca^2+^ in the center is modulated by diffusion and SR Ca^2+^ transport systems.

**FIGURE 2:**
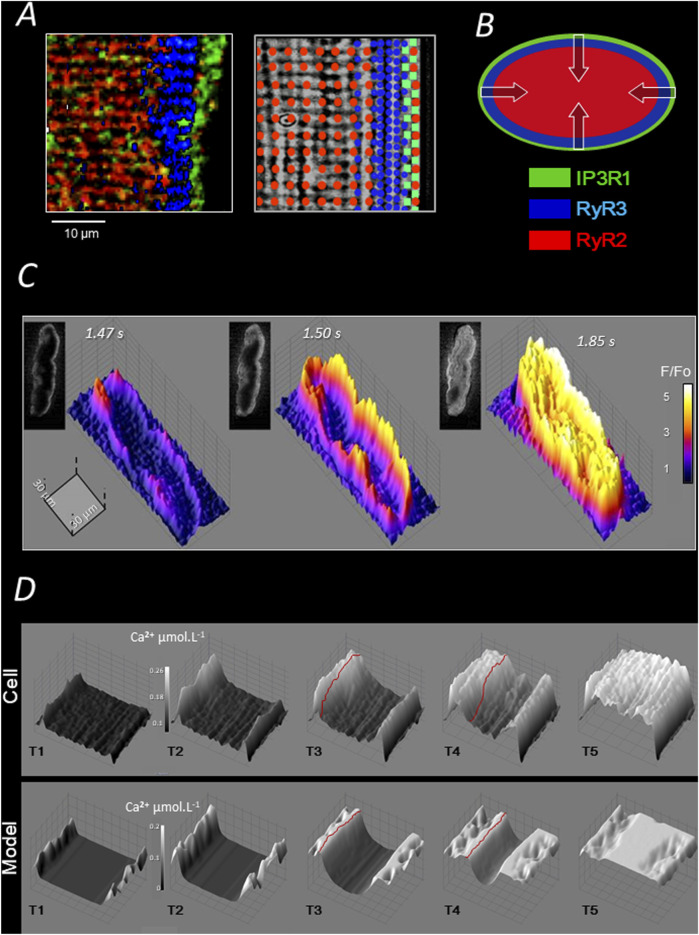
Ca^2+^ handling and electrically evoked Ca^2+^ mobilization of pig Purkinje cells. **(A)** Each of the three channel regions described in Figure 1B is stained with a specific antibody in the left panel (color legend is represented in B); the three regions partially overlap over a few micrometers; as shown in the right panel, the intermediate expression of RyR3 overlaps with both RyR2 and IP3R regions and some RyR2 are expressed in the IP3R region (Stuyvers et al., 2005); **(B)** the layered arrangement of SR Ca^2+^ channels shown in A allows for centripetal “layer-to-layer” activation by CICR and Ca^2+^ diffusion (Daniels et al., 2017). **(C)** 2D and 3D illustrations of three representative sequences of the (AP-mediated) centripetal Ca^2+^ propagation in pig Purkinje cells (see text). **(D)** Computational modelling of the centripetal propagation of Ca2+ in pig Purkinje cells; red lines underline the Ca^2+^ front moving toward the cell centre by CICR and diffusion (Haq et al., 2013).

In large mammalian species, the absence of T tubules reduces the total membrane surface area of the cell, which likely also contributes to the low total membrane resistance and rapid conduction of Purkinje fibers. The functional consequence of this structural particularity in Purkinje cells is a non-uniform and slower intracellular Ca^2+^ mobilization compared to that of ventricular cardiomyocytes (Boyden et al., 2003). A biphasic Ca^2+^ response to electrical stimulation was reported in Purkinje cells, based on the aequorin signal, by Wier 45 years ago (Gil and Hess, 1984). Consistently, using more recent Ca^2+^ probes and advanced Ca^2+^ imaging techniques, we found that stimulation induces a first release of Ca^2+^ under the sarcolemma, probably from junctional SR in the SubSL, followed by the progression of a front of elevated Ca^2+^ toward the cell center (Haq et al., 2013); see Figure 2. Sarcomere shortening is observed at the end of the progression, when the cytosol is fully loaded with Ca^2+^. A specific model of Ca^2+^ mobilization demonstrated that this “centripetal” propagation results from a combination of Ca^2+^ diffusion and consecutive Ca^2+^ release (by CICR) from concentric layers of the SR (Dun and Boyden, 2008; Boyden et al., 2010); Figure 2B. Presumably supporting this centripetal Ca^2+^ mobilization and, more generally, possibly compensating for the absence of T tubules, Purkinje cells were shown to express in canine heart three types of SR-Ca^2+^ channels (Daniels et al., 2017; Stuyvers et al., 2005) (Figures 1B, 2A): IP_3_R1 under the sarcolemma, RyR3 deeper but still in the peripheral region of the SR, and the “cardiac” RyR2 in the central SR. The distinct localization of these channels defines three specific regions of SR-Ca^2+^ release (Stuyvers et al., 2005). So far, we have observed the same arrangement of channels and the same centripetal Ca^2+^ mobilization in dog, sheep, pig, and human Purkinje cells.

In summary, as shown in Figure 2D, the electric stimulation of Purkinje cells mediates a typical centripetal Ca^2+^ mobilization, which is produced by the consecutive activation of IP3R1-, RyR3-, and RyR2-Ca^2+^ release regions. A computational model of this mechanism has been proposed in (Haq et al., 2013).

Like RyR2 in the ventricular myocytes, the three SR-Ca^2+^ release channels of the Purkinje cell spontaneously generate stochastic spark- and wave-like events (Stuyvers et al., 2005), as illustrated in Figure 1B. Because of different biophysical properties, the three channels open at distinct Ca^2+^ concentrations and with different kinetics, creating three regions of specific spontaneous Ca^2+^ events (Figure 1B); (Stuyvers et al., 2005; Daniels et al., 2017): large asymmetrical Ca^2+^ sparks under the sarcolemma, small Ca^2+^ waves (“wavelets”) that propagate over short distances in the peripheral RyR3-region, and large Ca^2+^ waves (“Cell-Wide-Waves” or “CWWs”) spanning the entire width of the cell and travelling in the cell and cell-to-cell in the longitudinal direction of the fibers. The CWWs are of sufficient magnitude to depolarize the membrane through NCX activation and are the Ca^2+^ events underlying the DADs in Purkinje cells (Boyden et al., 2000); see Panel B in Figure 1. Wavelets are thought to be the triggering events (by CICR) of the CWWs in the cell periphery (Stuyvers et al., 2005). Regular Ca^2+^ sparks were observed in the three regions of Ca^2+^ release (Stuyvers et al., 2005; Hirose et al., 2006).

### Ca^2+^ arrhythmogenicity of Purkinje fibers post infarction

Delayed afterdepolarization (DAD) is the electrical event that initiates triggered arrhythmias in the Purkinje fibers. DADs are observed in all cardiac cells and are caused by spontaneous rises in cytoplasmic Ca^2+^ concentration (Figure 1B), commonly resulting from SR Ca^2+^ release (Hirose et al., 2006), and, occasionally in cardiomyocytes, by sudden Ca^2+^ demobilization from the myofibrillar troponin C (Ter Keurs et al., 2006). The increase of cytosolic Ca^2+^ activates NCX, generating a forward mode I_NCX_ current, as well as other Ca^2+^−sensitive currents, such as chloride currents and non-selective cationic currents (Ter Keurs and Boyden, 2007). The resulting ionic imbalance depolarizes the membrane, generating a DAD. Technically, the amplitude of the DAD depends on the size of the resting K^+^ conductance, mainly determined by the inward-rectifier K^+^-current I_K1_, relative to I_NCX_ amplitude (Landstrom et al., 2017). A spontaneous AP can arise when the DAD amplitude reaches the threshold of the inward I_Na_ current (Figure 1B). The spontaneous APs in Purkinje fibers activate the surrounding myocardium, producing a premature ventricular beat/contraction (PVB/PVC), the first marker of a more severe tachycardic occurrence (Reder et al., 1981; Dosdall et al., 2007). The frequency and amplitude of DADs and PVBs determine the onset of the tachyarrhythmia in the ventricle.

Therefore, Ca^2+^ is the principal player in the generation of DADs, and the abnormal spontaneous Ca^2+^ activity in the Purkinje cells is widely recognized as a cause of tachyarrhythmias in the ischemic heart (Haissaguerre et al., 2016).

Although the fundamental alteration that leads to the “electrogenic” Ca^2+^ release in Purkinje cells remains unknown, several hypotheses can be discussed from our current understanding of Purkinje cells. First, inspired by the alteration of the RyR2 reported in inherited tachycardic diseases, such as CPVT (Priori and Chen, 2011; Herron et al., 2010; Kang et al., 2010), aberrant SR-Ca^2+^ release has been proposed to explain the arrhythmogenicity of Purkinje cells post-infarction (Hirose et al., 2006; Hirose et al., 2008). Similarly, an upregulation of SR-Ca^2+^ release by the reticular protein CASQ2 (Chen et al., 2013) or a reduction of its Ca^2+^ buffering capacity due, e.g., to an alteration similar to the mutation discussed in (Faggioni and Knollmann, 2012; Galimberti and Knollmann, 2011), could increase the Ca^2+^ liberation by the SR. Alternatively, a potentiation of SR-Ca^2+^ uptake would be expected to accelerate the transfer of Ca^2+^ from Ca^2+^ pumps to the Ca^2+^ release channels, possibly causing the abnormal increase in SR-Ca^2+^ release in Purkinje cells.

Beyond the alteration underlying the pro-arrhythmic augmentation of spontaneous Ca^2+^ release, another striking question is how ischemia in the ventricular myocardium induces the Ca^2+^ arrhythmogenicity in the Purkinje fibers.

It is well known that Ca^2+^ overload arises in cells directly exposed to ischemia, mainly due to the depletion of cellular energy and depression of ATP-dependent Ca^2+^ extrusion from the cytosol. In addition to loading the SR with Ca^2+^ and increasing the spontaneous SR-Ca^2+^ release (“SR Ca^2+^ leak”), the excess of Ca^2+^ in the cell triggers multiple reactions involved in apoptosis, hibernation, and cell death (Webster, 2012; Lüss et al., 1998). This is the case in ventricular myocytes of the infarction area. However, the early evidence of increased spontaneous Ca^2+^ activity in Purkinje cells after MI has been found in free-running Purkinje fibers spanning the subendocardial region. Except at the Purkinje-myocardium interface, these fibers are anatomically independent of the ventricular myocardium and are frequently connected to the endocardium outside the ischemic area. In this situation, it is logical to anticipate that these free-running strands primarily rely on O_2_ and nutrients from the surrounding blood flow in the chamber (Janse and Wit, 1989) and are not directly exposed to the ischemic conditions affecting the myocardium. Interestingly, this may suggest a potential release of bioactive (paracrine) agents by the ischemic myocardial cells and “remote” impact on the intracellular Ca^2+^ handling of subendocardial Purkinje strands.

Purkinje fibers are involved in many different types of cardiac arrhythmias, with a majority related to myocardial ischemia and infarction, as reviewed in (Nogami et al., 2023). The mechanisms of these arrhythmias evolve with the different stages of the MI, but the exact time course of Purkinje-mediated arrhythmicity from the onset of an acute ischemic attack to the healed MI and scar formation is not clearly established in human patients. In the dog model of coronary ligation, the Purkinje arrhythmogenicity has been reported under the form of Purkinje-mediated triggers of VTs and VFs after reperfusion during the acute phase 1b (∼30 min) (Xing and Martins, 2001; Arnar and Martins, 2002). After the scar formation, in the long-term chronic phase 3 (weeks, months) post MI, Purkinje fibers surviving in the healed MI area, remain excitable but more prone to Ca^2+^-mediated DADs. The Purkinje focal activity could occasionally trigger monomorphic VTs or participate in reentrant circuits in a minority of patients (less than 5%) (Bogun et al., 2006). Ablation of these foci usually abolishes those late and often recurrent arrhythmias (Charton et al., 2023). Nevertheless, animal models have shown that Ca^2+^-mediated arrhythmogenicity is a specific feature of Purkinje fibers located in the border zone of the infarct and arises during the subacute phase 2 (within 48–72 h) while the infarct is still evolving (Haissaguerre et al., 2016). Despite the known large prevalence of VTs and VFs, likely due to abnormal Ca^2+^ handling-induced DADs in Purkinje cells, and the high risk of sudden cardiac death during this phase (Frampton et al., 2023), no precise quantification of phase 2 arrhythmic incidence has been reported in humans (Sattler et al., 2019).

### Importance of animal models for the study of Ca^2+^ arrhythmogenicity

Animal models are indispensable for providing not only mechanistic insight into the Ca^2+^ arrhythmogenicity of Purkinje fibers in humans but also assisting the development of targeted antiarrhythmic therapies. Pursuing these goals requires high-resolution tools and invasive techniques that further justify the use of animal models of the human heart.

Nevertheless, the differences identified so far between Purkinje cells and ventricular myocytes have been observed in the hearts of large mammalian species. In small rodents, Purkinje cells are comparable to ventricular myocytes as both cell types contain the same arrangement of myofibrils, abundant transverse tubules, and well-developed sarcoplasmic reticulum (Di Maio et al., 2007). Interestingly, the presence of T tubules in mouse and rat Purkinje cells suggests that Ca^2+^ handling and Ca^2+^ mobilization in small rodents do not show the specific characteristics found in cells of large animals, potentially including humans. Supposing that these differences of Purkinje fibers compared to myocardium found in large-sized hearts are to facilitate the conduction, their absence in mice and rats is not surprising, since the impulse propagation distance is shorter and, therefore, the need for low-resistance fibers is less than in larger hearts.

To date, experimental and clinical data support the conclusion that the post-MI risk of ventricular fibrillation and cardiac arrest in humans is linked to a deficient component of Ca^2+^ handling in Purkinje cells (Haissaguerre et al., 2016). Models addressing this deficiency must consider the interspecies differences, specifically the discrepancy in Ca^2+^ handling systems and Ca^2+^ mobilization between small and large animals. For instance, the hypothesis of aberrant SR Ca^2+^ release as the source of arrhythmogenicity may ultimately apply to mouse or rat models in which Purkinje cells are likely to express only one SR Ca^2+^ channel (RyR2). The same hypothesis is less likely in large animal species. Increased SR-Ca^2+^ release has been evidenced 48 h after coronary ligation in canine Purkinje cells, in the three regions expressing distinct channels (Hirose et al., 2006). The simultaneous alteration of the three channels in distinct subcellular regions within 2 days is improbable. As another example, the IP_3_R1 channel has been recently implicated in the arrhythmogenesis of the human heart (Sun et al., 2025). However, confirmation of this implication has been achieved by inducing IP3R1 expression in a mouse model of Purkinje cells, which are expected to express a radically different Ca^2+^ handling system compared to that predicted in human Purkinje cells, where IP3R1 is likely to play a role in the centripetal Ca^2+^ mobilization.

Our current knowledge of Purkinje cells in large animal species strongly suggests that the subcellular foundations of Ca^2+^ arrhythmogenicity differ between mouse and human Purkinje cells. Considering the arguments supporting abnormal Ca^2+^ handling as a probable source of Purkinje pro-arrhythmicity in ischemic human hearts, only cell models with a Ca^2+^ management system consistent with that of common large mammalian species will be suitable for identifying the molecular origins of triggered arrhythmias in humans.

In addition, the anatomy of the Purkinje tissue influences the cardiac conduction (Vigmond and Stuyvers, 2016) and also displays notable differences among animals. For example, horses have more abundant and thicker Purkinje fibers compared to dogs. Like human and rabbit Purkinje fibers, most dog Purkinje fibers extend in the subendocardial region as free strands with many subendocardial connections with the myocardium. On the contrary, most pig Purkinje fibers are transmural and connect with the myocardium throughout the ventricular wall (Gómez-Torres et al., 2021; Šolc, 2007; Lelovas et al., 2014). Overall, the pig heart is currently recognized as one of the most effectiv translational models for cardiovascular diseases (Mackenzie et al., 2004; Stuyvers et al., 2005), while the dog heart is widely used in cardiac electrophysiology (Willis and Oliveira, 2018). Nevertheless, the Purkinje cells of both species appear to exhibit the same features currently predicted in human cells. This aspect may also be considered when selecting translational models for ischemic arrhythmias.

Finally, while murine models are well adapted to study a specific protein expression the distinctive features of the heart, including the Purkinje system, in the small animals should be considered in the mechanistic studies of Purkinje-induced Ca^2+^ arrhythmia in humans.

## Conclusion

Purkinje fibers, once considered passive conductors, are now clinically recognized as active arrhythmogenic agents in the ischemic and infarcted heart. Animal models strongly suggest that the unique Ca^2+^ handling of Purkinje cells is involved in the spontaneous depolarizations that initiate lethal ventricular tachyarrhythmias upon ischemic myocardial infarction. The discovery of specific features in the Ca^2+^ handling of Purkinje cells is relatively recent. Numerous questions remain concerning the evoked and spontaneous activation of these cells in the current translational animal models, and the applicability of many findings to humans, although highly probable, has not yet been formally established. However, the level of our knowledge is sufficient to indicate that the choice of animal models for human ischemic arrhythmias must integrate the unique features recently discovered in the Purkinje cells of large mammalian species.

Intracellular Ca^2+^ manipulations have already been considered for treating the ischemic arrhythmic risk (Ter Keurs and Boyden, 2007; Boyden and Ter Keurs, 2005). Still, the lethal arrhythmias will remain unpredictable as long as the exact origin of Ca^2+^ dysfunctions in Purkinje fibers of ischemic heart is not clarified.
